# Might nontransferrin-bound iron in blood plasma and sera be a nonproteinaceous high-molecular-mass Fe^III^ aggregate?

**DOI:** 10.1016/j.jbc.2022.102667

**Published:** 2022-11-09

**Authors:** Shaik Waseem Vali, Paul A. Lindahl

**Affiliations:** 1 Department of Biochemistry and Biophysics, Texas A&M University, College Station, Texas, USA; 2 Department of Chemistry, Texas A&M University, College Station, Texas, USA

**Keywords:** Mössbauer spectroscopy, hereditary hemochromatosis, β thalassemia, desferrioxamine, LC-ICP-MS, electron paramagnetic resonance, ferroportin, hepcidin, DFO, deferoxamine, EPR, electron paramagnetic resonance, FPN, ferroportin, FTN, ferritin, FTS, flow-through solution, *HFE*, Homeostatic Fe regulator gene, commonly mutated in hereditary hemochromatosis, ICP-MS, inductively coupled plasma mass spectrometry, LC, liquid chromatography, LMM, low molecular mass, LN2, liquid nitrogen, MB, Mössbauer spectroscopy, R24 and R36, 24 and 36 weeks-old mice raised on a regular/standard diet, TFN, transferrin

## Abstract

The *HFE* (Homeostatic Fe regulator) gene is commonly mutated in hereditary hemochromatosis. Blood of (*HFE*)(−/−) mice and of humans with hemochromatosis contains toxic nontransferrin-bound iron (NTBI) which accumulates in organs. However, the chemical composition of NTBI is uncertain. To investigate, *HFE*(−/−) mice were fed iron-deficient diets supplemented with increasing amounts of iron, with the expectation that NTBI levels would increase. Blood plasma was filtered to obtain retentate and flow-through solution fractions. Liquid chromatography detected by inductively coupled plasma mass spectrometry of flow-through solutions exhibited low-molecular-mass iron peaks that did *not* increase intensity with increasing dietary iron. Retentates yielded peaks due to transferrin (TFN) and ferritin, but much iron in these samples adsorbed onto the column. Retentates treated with the chelator deferoxamine (DFO) yielded a peak that comigrated with the Fe–DFO complex and originated from iron that adhered to the column in the absence of DFO. Additionally, plasma from younger and older ^57^Fe-enriched HFE mice were separately pooled and concentrated by ultrafiltration. After removing contributions from contaminating blood and TFN, Mössbauer spectra were dominated by features due to magnetically interacting Fe^III^ aggregates, with greater intensity in the spectrum from the older mice. Similar features were generated by adding ^57^Fe^III^ to “pseudo plasma”. Aggregation was unaffected by albumin or citrate at physiological concentrations, but DFO or high citrate concentrations converted aggregated Fe^III^ into high-spin Fe^III^ complexes. Fe^III^ aggregates were retained by the cutoff membrane and adhered to the column, similar to the behavior of NTBI. A model is proposed in which Fe^II^ entering blood is oxidized, and if apo-TFN is unavailable, the resulting Fe^III^ ions coalesce into Fe^III^ aggregates, a.k.a. NTBI.

Nontransferrin-bound iron (NTBI) is a toxic form of iron in the blood of individuals with iron-overload diseases such as hereditary hemochromatosis (1) and β-thalassemia (2). It accumulates excessively in the liver and other organs, resulting in liver fibrosis, cirrhosis, cancer, endocrinopathies, and cardiomyopathies (3). Treatments include low iron diets, frequent phlebotomies, and ingestion of iron-binding chelators. Effectiveness is limited, especially for β-thalassemia.

Transferrin (TFN) is an iron-binding protein in the blood which serves as an iron buffer (4). In healthy individuals, about one-third of TFN is in the holo- (two Fe^III^ ions bound) form whereas most of the remainder is apo- (iron-free). Nutrient iron enters the blood *via* ferroportin (FPN), a membrane-bound Fe^II^ transporter that is highly expressed on the basolateral side of enterocytes in the duodenum (5). Holo-TFN is distributed throughout the body and enters cells *via* TFN receptor–mediated endocytosis. FPN is also highly expressed in macrophages, including Kupffer cells in the liver and red-pulp macrophages in the spleen. In both cases, FPN functions to release stored iron into the blood.

Iron import into the body is regulated at the systems’ level by hepcidin, a small peptide hormone produced in the liver in response to excessive bodily iron (5). Hepcidin binds FPN, causing its internalization and subsequent hydrolytic degradation. Individuals with hemochromatosis, most commonly harboring a mutation in the *HFE* (Homeostatic Fe regulator) gene, generate insufficient hepcidin, resulting in excessive levels of FPN and thus excessive import of nutrient iron into the blood. This saturates TFN such that the concentration of apo-TFN available to receive newly imported iron is insufficient. The excessive iron released into the blood becomes NTBI.

Despite being recognized to exist for a half-century, the chemical identity of NTBI remains unestablished (1, 2, 3, 4, 5, 6, 7, 8, 9, 10, 11, 12, 13, 14). One reason for this is that its two most predominant characteristics—accumulating in organs and susceptibility to chelation—are indirect and difficult to quantify. Iron accumulates in organs primarily as ferritin (FTN), but NTBI is a different species that is likely altered *via* reduction and ligand exchange as it enters the cell and is converted into FTN. NTBI is typically defined operationally as the iron in plasma that reacts with a particular chelator under prescribed concentrations and durations. However, the size of the NTBI pool in plasma is affected by these details. A second problem is that the concentration of NTBI in diseased plasma is not exceptionally high (1–10 μM), and although NTBI concentration in healthy individuals is lower, it is still detectable and significant. A third problem arises from the gradual and ambiguous “spillover” conditions required to generate NTBI; between 40% and 70% TFN saturation is reportedly sufficient for NTBI levels to increase (10, 15, 16). A fourth problem is that the aqueous redox and coordination chemistry of iron is complicated, and NTBI may be heterogeneous (12, 17).

Two basic types of experiments have contributed to our understanding of NTBI, but perhaps they have also hindered it. One type of experiment has been to monitor the fate of added radioactive ^59^Fe to sera or plasma. Radioactive iron binds preferentially and tightly to available apo-TFN, and the excess is concluded to be (or become) NTBI. This assumes that the only iron-binding ligand in sera/plasma is the NTBI ligand (symbolized :L_NTBI_) and that this ligand is present in excess. Actually, there are many potential ligands in plasma (18) and the added ^59^Fe may bind any or all of them.

In the other type of experiment, a chelator is added to sera/plasma, and the resulting Fe–chelator complex is assumed to arise from the binding of NTBI; the assumed general reaction is {Fe:L_NTBI_ + chelator ⇄ Fe-chelator +:L_NTBI_}. The problem is that NTBI is destroyed during this reaction, making it unlikely that such experiments can ultimately be used to identify NTBI. Moreover, the added chelator might also sequester iron that is bound to other non-NTBI species, overestimating the size of the NTBI pool.

To avoid these problems, we and others (19) have investigated *untreated* blood using liquid chromatography (LC) interfaced to an online inductively coupled plasma mass spectrometer (LC-ICP-MS), and have detected and characterized endogenous iron-containing complexes without adding iron or chelators. Neu *et al.* (19) detected Fe^III^(citrate) in human blood plasma by ESI-MS, supporting the conclusion that NTBI = Fe^III^ citrate. Dzubia *et al.* (20) detected low concentrations of low-molecular-mass (LMM) iron species in plasma from healthy humans, horses, mice, and pigs, but the chromatographic properties of these species largely differed from those of Fe^III^(citrate). Unexpectedly, the 10 kDa plasma ultrafiltrate (or flow-through solution, FTS) from human hemochromatosis patients did not exhibit *any* additional iron-detected LC peaks relative to healthy controls. However, the patients had been treated for the disease, suggesting that their NTBI levels might have been too low to detect.

Dzubia *et al.* (21) hypothesized that the low concentration of LMM iron species observed in the earlier study was due to the quantitative removal of NTBI by the liver, and so they surgically implanted catheters in the portal vein of pigs that had been starved for iron. Intestinal blood passes through this vein to the liver, and so they anticipated that blood removed from it would contain high concentrations of NTBI. Blood was also removed from the caudal/cranial vena cava as a control. A bolus of ^57^Fe was injected into the stomach *via* a feeding tube, and blood samples were removed from both catheters at increasing times. Since only 2% of natural-abundance iron is due to ^57^Fe, the fate of the injected enriched ^57^Fe could be followed. Surprisingly, the LMM iron complexes did not become enriched in ^57^Fe; rather the injected ^57^Fe bound apo-TFN. Dzubia *et al.* (21) concluded that the detected LMM iron complexes arise from internal stores rather than directly from nutrient iron.

Building off those results, we hypothesized that NTBI might only be detectable using iron-overloaded genetically modified animals. Here, we investigated this by examining *HFE*(−/−) (heretofore called *HFE*) mice fed iron-deficient diets supplemented with 0, 50, 500, and 5000 mg of natural abundance Fe^III^ citrate. We selected the *HFE* gene because a mutation in it is the most common origin of the disease (22). Using our LC-ICP-MS system, we expected to observe increasing levels of LMM iron complexes in the blood plasma filtrates of these sick animals, especially as the concentration of iron in the diet increased. Once again, our results were unexpected, but they prompted a new and intriguing hypothesis as to the chemical nature of NTBI. Figure 1 outlines the chronology of our study.

**Figure 1:**
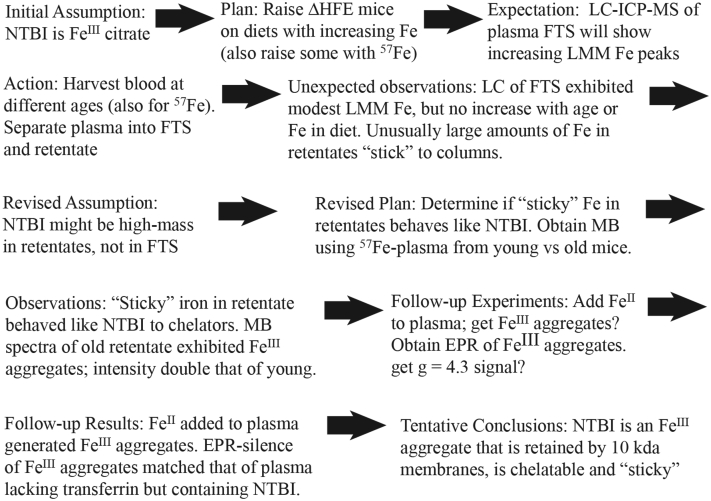
Timeline for study.

## Results

Our initial objective was to detect and characterize NTBI in the blood of *HFE* mice (and controls) raised on 0, 50, 500, and 5000 mg of iron per kg of iron-deficient chow. Some *HFE* mice were raised on 50 mg ^57^Fe/kg chow. Mice were sacrificed at various ages, blood was collected, and the plasma portion was filtered through a 10 kDa cut-off membrane, resulting in retentate and FTS fractions. A flow chart showing an overview of the study is given in Fig. S1.

We initially focused on detecting NTBI in FTSs since we expected it to be a LMM species. FTSs of the plasma from both *HFE* and control mice were subjected to LC-ICP-MS chromatography using a size-exclusion low-mass column that could resolve species with masses ranging from 100 to 10,000 Da. In a typical experiment performed over the course of a day, eight *HFE* or control mice were sacrificed, two from each diet group. Adult mice yielded only 0.5 to 1 ml of blood, and so blood of two mice from each group (3 for younger mice) were combined. After centrifugation, plasma fractions, representing ∼ 50% of total volume, were collected and filtered, yielding ∼ 70 μl of retentate and ∼ 400 μl of FTS.

The iron concentration in *HFE* FTS (from six 12–16 weeks mice) was modest, 1.9 ± 0.2 μM Fe. Plasma contains hundreds of mM of salt which fouled the ICP-MS instrument and reduced the detector response. Significant amounts of iron adsorbed onto the column, and subsequent cleaning had limited effectiveness. Small differences in daily tuning of the ICP-MS and changes in columns caused shifts in peak intensities and/or elution volumes. As a result, traces obtained on different days were not easily compared, so analyses were limited to assessing overall patterns *within a group of traces obtained on the same day*.

**Figure 2:**
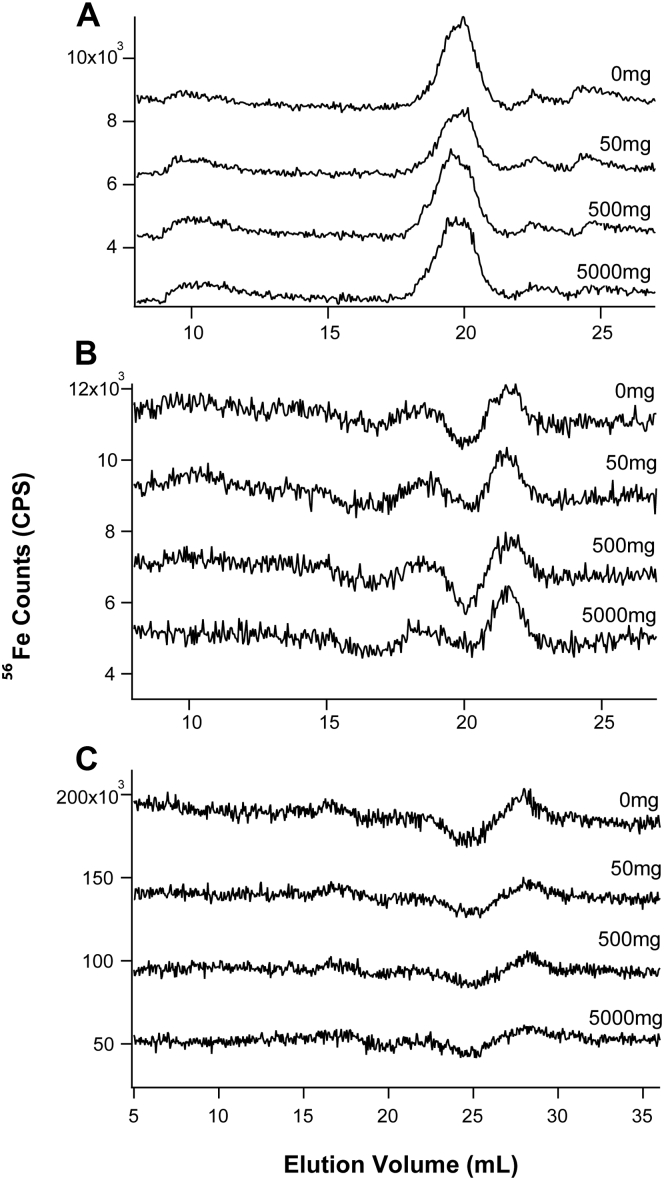
**Iron-detected chromatograms of plasma FTSsrun on the low-mass column.***Panel A*, 3 weeks *HFE* mice. *Panel B*, 12 weeks *HFE* mice. *Panel C*, 16 weeks control mice. Food group of mice are shown in the figure. FTS, flow-through solution; *HFE*, homeostatic Fe regulator gene commonly mutated in hereditary hemochromatosis.

Chromatograms of FTS from *HFE* and control mice exhibited 1 to 4 low-intensity iron peaks (Fig. 2). Results of three separate experiments are shown, including from 3-weeks *HFE* mice (Panel A), 12 weeks *HFE* mice (Panel B), and 16 weeks control mice (Panel C). Iron-detected traces from four additional experiments are shown in Fig. S2, including FTS from 3 weeks *HFE* (Panel B), 24 weeks *HFE* (Panel C), 32 weeks *HFE* (Panel A), and 24 weeks control (Panel D) mice. Specific peaks *within* a group were nearly identical in terms of elution volumes and intensities. Peaks in SI experiments were more intense; however, some of that intensity was due to contaminating iron that had desorbed from the column with each sample injection. In the experiment of Fig. S2*D*, we verified this by running a “ghost” column (*i.e.*, peek tubing in place of the column) and measuring the area under the resulting iron peaks. Although this indicated substantial contaminating iron in LC traces, the nearly identical intensity of the ghost column peaks confirmed that the concentration of iron in the FTS did NOT increase proportionately with dietary iron. All such experiments indicated the same.

In the experiment of Fig. S2*C*, an Fe^III^ citrate standard run on the same day did not comigrate with the Fe peaks from plasma, supporting our previous conclusion (20, 21) that Fe^III^ citrate was not the dominant LMM Fe species in plasma FTS. The LMM iron species in the FTS of *HFE* (or control) mice were present at low concentrations and were similar regardless of whether the sample originated from *HFE* or control mice and regardless of the iron level in the diet. Overall, these results forced us to conclude that *plasma FTS from HFE mice do not accumulate a LMM form of iron even on a high-iron diet*.

**Figure 3:**
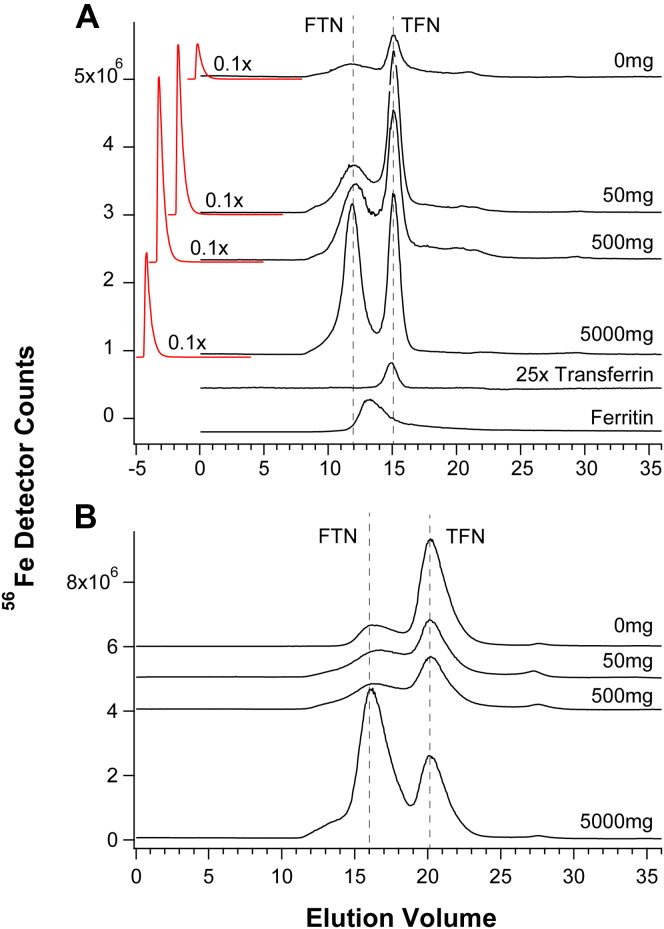
**Chromatograms of plasma retentates onthehigh-mass column.***Panel A*, 24-weeks-old *HFE* mice. Flow rate was 0.6 ml/min. Matching ghost column traces (×0.1) are in *red*. Also shown are traces of ferritin and TFN (×25). Ferritin was from horse spleen which may have resulted in a slight shift in elution volume. *Panel B*, 24-weeks-old control mice. Flow rate was 0.5 ml/min. FTN, ferritin; *HFE*, homeostatic Fe regulator gene commonly mutated in hereditary hemochromatosis; TFN, transferrin.

With some reluctance, we shifted the search for NTBI to retentate fractions. Such fractions from 24 weeks control and *HFE* mice were run on a high-mass column which resolved species with masses between 10 kDa and 100 kDa (Fig. 3, *A* and *B*). Matching ghost column traces (×0.1) are in red. Two major peaks were observed, which were assigned to FTN and TFN by running protein standards of horse spleen FTN (Sigma) and human TFN (Athens Research). The pattern in Fig. 3*A* suggested that the TFN peak intensity in *HFE* retentates increased from 0 to 50 mg Fe and then remained relatively constant. The peak assigned to FTN increased gradually with dietary iron. For control retentates, the FTN peak also increased with dietary iron (Fig. 3*B*). No such trend was evident for TFN. Other batches of retentate analyzed by LC-ICP-MS showed increasing TFN and FTN saturation with increasing Fe in the diet. Ghost column areas in Fig. S3*A* indicated little contamination; however, 35% to 70% of sample iron typically adsorbed on the column. Retentates from control and *HFE* mice were devoid of LMM species (Fig. S4). The intensity of the void volume peaks increased with increasing nutrient iron, especially for the *HFE* samples.

**Figure 4:**
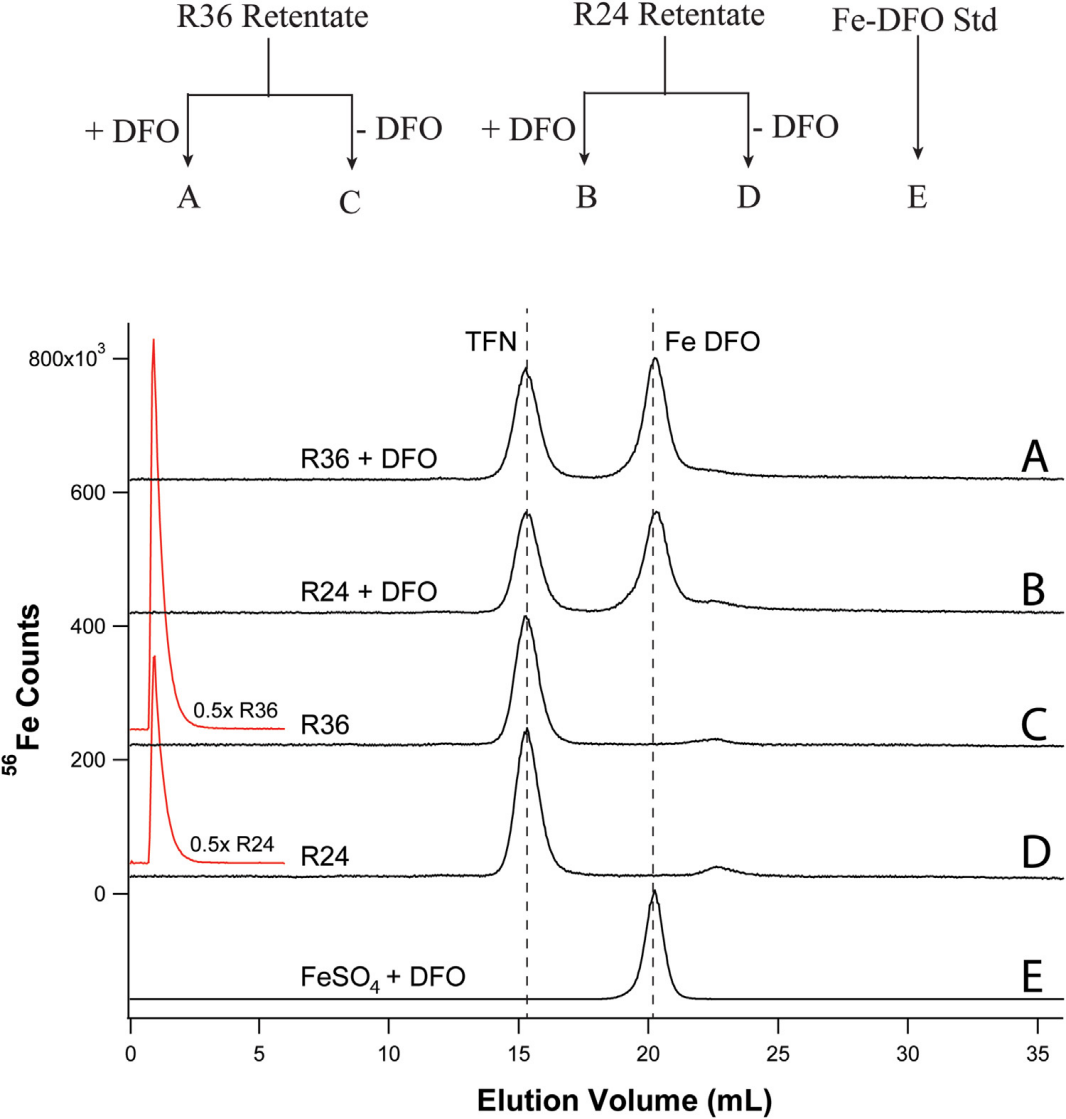
**Chromatograms of retentates with/without DFO on high-mass column.** A, R36 (“regulars”, 36 weeks) retentate was diluted 80× and treated with 10 μM DFO for 1 h; B, R24 retentate treated similarly; C, untreated R36 retentate diluted 80×; D, untreated R24 retentate; E, Fe-DFO standard (2 μM FeSO_4_ + 10 μM DFO). Corresponding ghost column traces (0.5×) are in *red*. Shown above is a diagram outlining this experiment. DFO, deferoxamine.

NTBI is commonly quantified by its reaction with deferoxamine (DFO), and so we treated retentate samples with this chelator to assess the presence of NTBI in those fractions. Some *HFE* mice were raised on a “regular” diet containing ∼250 mg iron/kg chow. Retentate samples from 24 to 36 weeks “regular” mice (called R24 and R36) were diluted 80-fold and divided in halves. Half of each sample was treated with DFO, and half was left untreated. All four samples were run on the high-mass and ghost columns (Fig. 4). A trace of an Fe-DFO standard was also collected. The dominant Fe-detectable peak for both R24 and R36 mice originated from TFN, and its intensity was similar for all four traces. Traces from the two samples that had been treated with DFO exhibited a second major peak which comigrated with the Fe-DFO standard. The intensity of these peaks did not increase at the expense of the TFN peak, rather the peaks simply appeared. We concluded that *HFE retentates contain a “sticky” form of iron that adsorbed onto the column but was also chelatable by DFO*. We will ultimately conclude that this material is NTBI. The corresponding ghost column peaks indicated that the R36 retentate contained more than twice as much iron as the R24 retentate, suggesting that the DFO-chelatable iron increased with age, as expected for NTBI.

**Figure 5:**
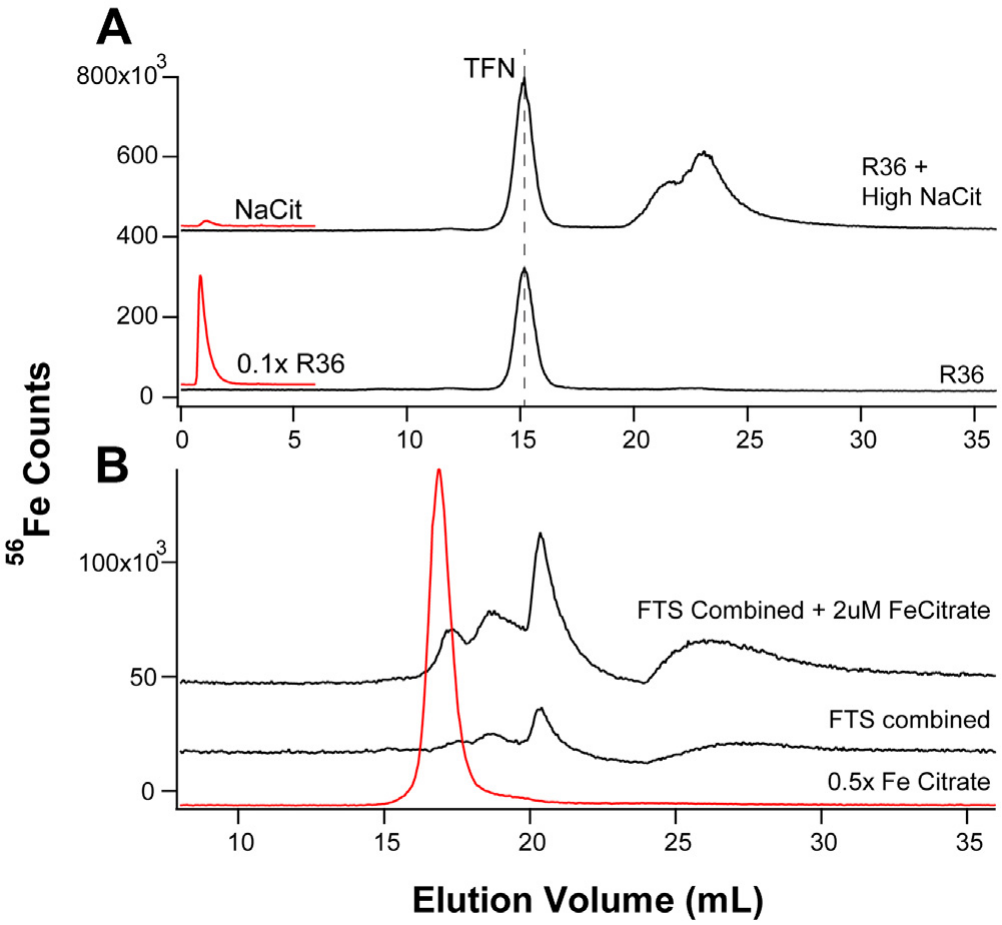
**Chromatograms of plasma retentates and FTSs with added citrate on high-mass and low-mass columns.** Panel *A*, (high-mass column) washed and diluted plasma retentate from 36-weeks-old “regular” *HFE* mice. Top chromatogram, after treatment with 50 μM Na citrate; bottom before treatment. *Panel B*, (low-mass column) FTS from combined 24 to 36 weeks 0, 50, 500, and 5000 mg *HFE* mice. Top chromatogram, after adding 2 μM Fe^III^ citrate. Middle chromatogram, before treatment. Bottom trace, Fe^III^ citrate standard (2 μM FeSO_4_ + 20 μM Na citrate). FTS, flow-through solution; *HFE*, Homeostatic Fe regulator gene commonly mutated in hereditary hemochromatosis; TFN, transferrin.

Citrate is the most popular candidate for the NTBI ligand, and so we performed experiments to explore this possibility. We attempted to remove LMM species from plasma, including Fe^III^ citrate, by filtering plasma from 36-weeks *HFE* “regular” mice using a 10 kDa cutoff membrane and washing the retentate twice with water. The retentate was then divided in two, half was treated with citrate, and half was untreated. Both halves were passed through the high-mass column. In both traces, the dominant peak was TFN (Fig. 5 Panel A). Quantification of the ghost-column trace indicated that 40% of the iron in the washed untreated retentate sample was not bound to TFN and was not detected in the trace. This undetected form of iron must have adsorbed to the column (ultimately, we will assign it to NTBI). The citrate-treated sample exhibited new peaks in the LMM region (at ∼ 22 ml) with approximately the intensity expected if citrate coordinated the high-mass “sticky” (NTBI) iron that was undetected in the other trace. We write this as {Fe^III^:L_NTBI_ + citrate → Fe^III^citrate +:L_NTBI_}.

Two similar experiments using plasma FTSs were analyzed using the low-mass column. In one experiment, FTSs from plasma of six mice were combined and then divided in two. Half was treated with an Fe^III^ citrate solution (which contained an excess of citrate), and half was untreated. An Fe^III^ citrate standard was also run. The trace of the untreated half exhibited ∼ 3 resolvable peaks at 16 to 21 ml elution volume plus a broad peak at longer elution volumes (Fig. 5*B*, middle trace). The trace of the treated sample (upper trace) was qualitatively similar but more intense. It included a peak that nearly comigrated with the Fe^III^ citrate standard (perhaps the slight shift was due to the salt present only in the FTS sample), but the intensity of the “Fe^III^ citrate” peak was modest relative to the increased intensities of the other peaks in the trace. (Note: the elution volume of Fe^III^ citrate that migrated through the low-mass column differed from that through the high-mass column.) We concluded that most (∼90%) of the iron from the added Fe^III^ citrate underwent ligand exchange with other species in the FTS. This suggested that Fe^III^ citrate is not the most stable iron complex formed when citrate is added to plasma; much of it converts to other forms resulting in a distribution of species.

If NTBI was present in retentate fractions rather than in FTSs, we realized that it might be possible to detect using Mössbauer spectroscopy (MB). With a limited amount of ^57^Fe-enriched plasma available from a recent study (23), we decided to combine plasma from the four youngest *HFE* mice that were available (4, 6, 10, and 14 weeks), concentrate the sample using a 10 kDa cutoff membrane, and load it into a MB cup for analysis. We did the same for plasma from four older *HFE* mice (18, 24, 32, and 52 weeks). Since no ^57^Fe-enriched plasma from control mice were available to serve as a control, we anticipated that NTBI might be present in greater amounts in the old mice sample.

The iron concentrations in the young and old retentates (after removing residual red blood cell contributions) were 96 ± 6 μM and 136 ± 5 μM, respectively. Since the retentates were concentrated ∼ 5-fold for this experiment, we estimate that the plasma iron concentration would be ∼ 20 μM in young and ∼ 27 μM in old plasma, similar to our previous values (20, 21).

**Figure 6:**
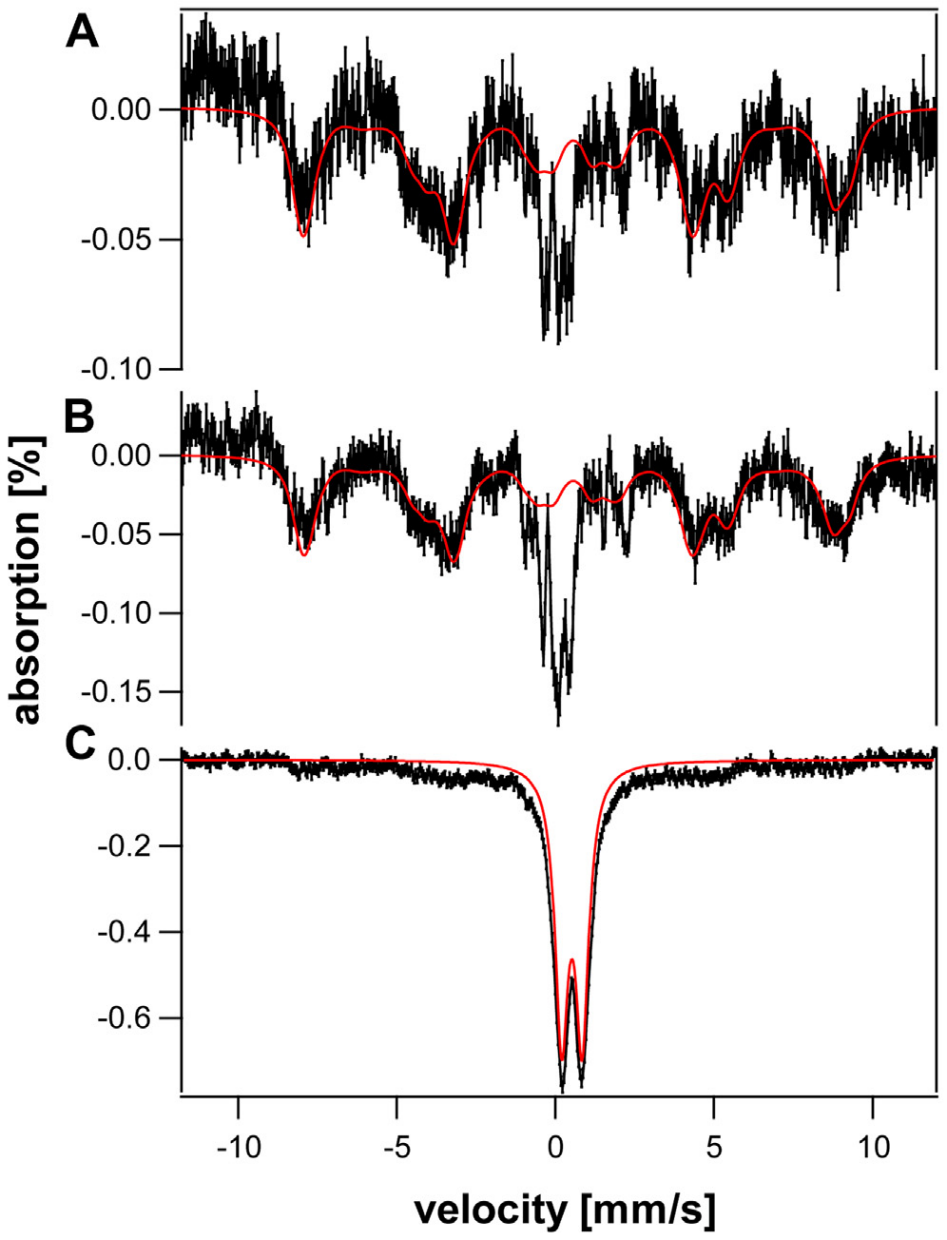
**5.5 K 0.05 T Mössbauer spectra of plasma retentates from young *versus* old**^**57**^**Fe-enriched *HFE* mice.***A*, young; *B*, old; *C*, 150 μM ^57^FeCl_3_ added to blood plasma from 24 weeks mice fed on regular diet not enriched in ^57^Fe. The *red lines* in *A* and *B* are simulations for the feature due to transferrin (S = 5/2; D = −0.25 cm^-1^; E/D = 0.30; ΔE_Q_ = 0.40 ± 0.02 mm/s; δ = 0.56 ± 0.02 mm/s; η = -1.5; A_iso_ = −30.18 MHz; Γ = 0.70 mm/s). The *red line* in *C* is a simulation assuming ΔE_Q_ = 0.60 mm/s, δ = 0.55 mm/s, and Γ = 0.45 mm/s. *HFE*, Homeostatic Fe regulator gene commonly mutated in hereditary hemochromatosis.

The dominating features in the raw 6 K 0.05 T MB spectra (Fig. S5) were quadrupole doublets originating from contaminating deoxy and oxy Fe^II^ hemoglobin; these were simulated and removed. The resulting difference spectra were dominated by a magnetic feature which was simulated using the spin Hamiltonian parameters of di-ferric TFN (24). The only remaining spectral feature (Fig. 6, *A* and *B* respectively) was narrow absorption in the central region. Spectral noise was significant even after > 200 h of data collection. Spectral noise was too severe to firmly assign the absorption in the central region, but it could be approximately simulated by a quadrupole doublet with δ = 0.5 ± 0.1 mm/s and ΔE_Q_ = 0.5 ± 0.1 mm/s. The parameters approximated those of Fe^III^ aggregates such as the oxyhydroxide phosphate–associated nanoparticles found in diseased mitochondria (δ = 0.52 mm/s and ΔE_Q_ = 0.63 mm/s; (25)). The intensity of this material was ∼ 2 × higher in the spectrum obtained from old *versus* young HFE mice. In animals with iron-overload diseases, NTBI concentration increases with age (26).

**Figure 7:**
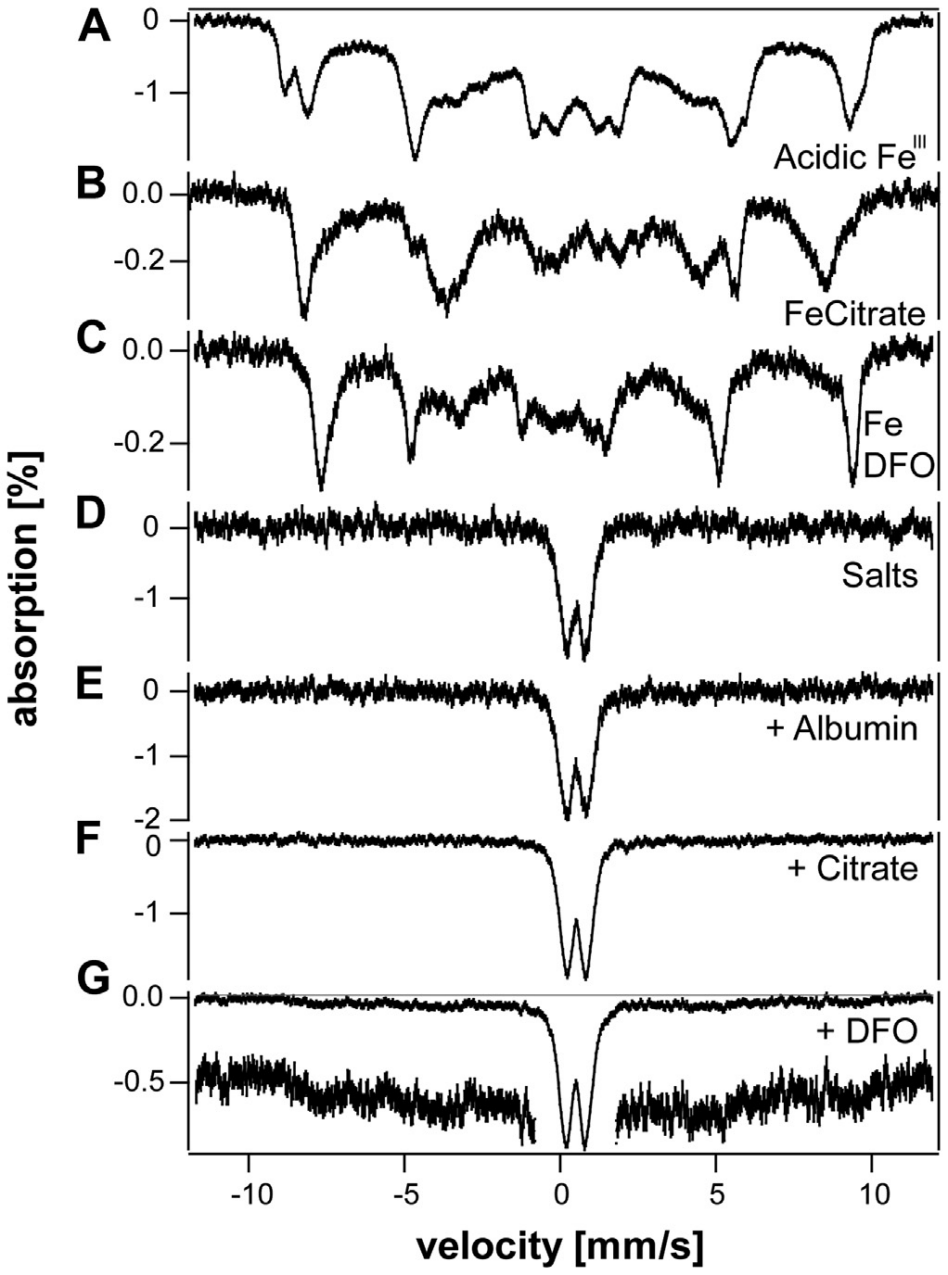
**5.5 K 0.05 T Mössbauer spectra of iron standards and pseudo-plasma.***A*, 250 μM acidic ^57^Fe^III^; *B*, same as A but mixed with 150 μM sodium citrate; *C*, same as A but mixed with 500 μM DFO; *D*, same as A but mixed with a pseudo-plasma salts; *E*, same as D plus 0.2 g/L bovine serum albumin; *F*, same as D plus 150 μM citrate; *G*, same as D plus 400 μM DFO (affording 200 μM ^57^Fe). The *red line* indicates baseline, and the *expanded region* highlights the magnetic features. DFO, deferoxamine.

Based on these observations, we hypothesized that *NTBI was an Fe*^*III*^ *aggregate in plasma*. We considered that such aggregates formed due to the oxidizing, salty, pH-neutral conditions of plasma. To consider this further, we added acidic ^57^Fe^III^ ions dropwise to blood plasma (to 250 μM) from 24 weeks *HFE* mice that had been fed a regular natural abundance-iron diet. The resulting spectrum (Fig. 6*C*) exhibited a broad quadrupole doublet again typical of ^57^Fe^III^ aggregates (simulated in red). The minor magnetic species evident from the baseline was probably from added ^57^Fe that bound apo-TFN. We next prepared a “pseudo plasma FTS” solution and added acidic ^57^Fe^III^ in similar fashion. The corresponding MB spectrum (Fig. 7*D*) was once again a quadrupole doublet with similar parameters (δ = 0.55 ± 0.02 mm/s and ΔE_Q_ = 0.60 ± 0.03 mm/s). In contrast, control spectra of acidic Fe^III^, Fe^III^ citrate, and Fe^III^-DFO were typical of magnetically isolated nonaggregated mononuclear high-spin Fe^III^ (Fig. 7, *A–C*).

Albumin and citrate are popular candidates for NTBI ligands, and both are present in plasma. Including either candidate in pseudo-plasma FTS had no effect on the resulting Mössbauer spectra (Fig. 7, *E* and *F*). In another experiment, we added DFO (Millipore) to the pseudo-plasma FTS that contained the Fe^III^ aggregate. In this case, the resulting Mössbauer spectra (Fig. 7*G*) revealed some coordination of Fe^III^ to DFO indicating that the aggregate can be partially broken up by the DFO chelator. Perhaps additional coordination would have been observed If incubation conditions had been optimized.

**Figure 8:**
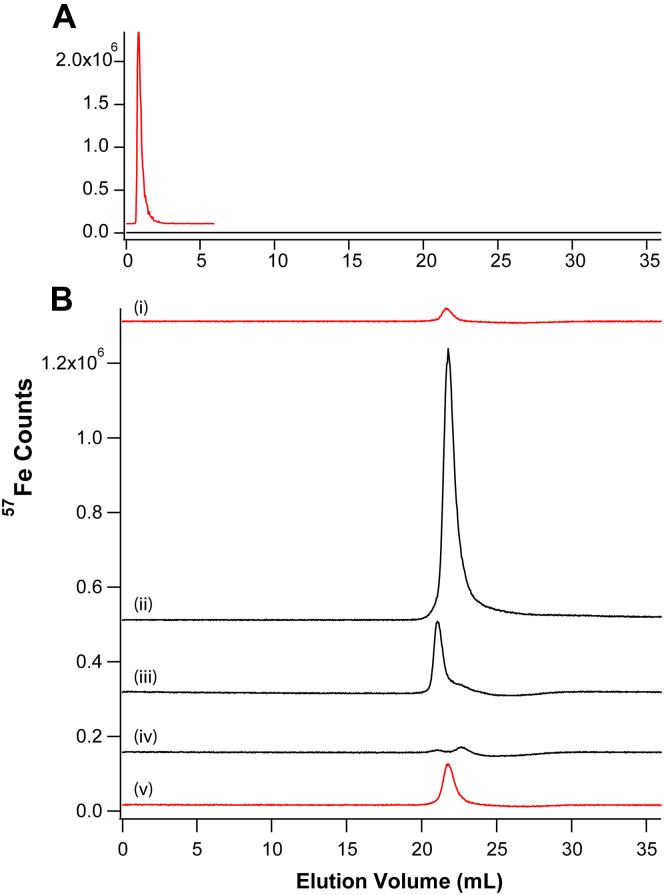
**LC-ICP-MS analysis of**^**57**^**Fe**^**III**^**aggregate in pseudo-plasma.***Panel A*, retentate from sample used in Figure 7*E* after being diluted 80× and analyzed using the high-mass column. *Red chromatogram*, same sample run through a ghost column. *Panel B*, trace: *i*, 150 μM sodium citrate run on a cleaned column; *ii*, 3 μM ^57^Fe^III^ citrate (150 μM citrate); *iii*, 3 μM of ^57^Fe^III^ plus pseudo-plasma salts plus 150 μM sodium citrate; *iv*, 3 μM ^57^Fe^III^ plus pseudo-plasma salts; *v*, same as *i* but after the other samples were run. Samples were incubated aerobically for 1 h prior to analysis. ICP-MS, inductively coupled plasma mass spectrometry; LC liquid chromatography.

To determine whether aggregated iron in the pseudo plasma behaved like the retentate of mice plasma, we filtered the sample of Figure 7*E* using the 10 kDa cutoff membrane. The resulting retentate was diluted, passed through the SEC column, and the resulting LC traces were analyzed like those of mice plasma retentates. All the aggregated Fe^III^ evident in the ghost trace shown in red adsorbed onto the column (Fig. 8, Panel A). Thus, the aggregated Fe^III^ material prepared in pseudo-plasma was “sticky”, a property shared with NTBI.

This property was modestly affected by including 150 μM sodium citrate in pseudo plasma salts containing 3 μM Fe. After incubating the samples 1 h aerobically, samples with and without citrate were analyzed by LC-ICP-MS (Fig. 8*B*). In the absence of citrate, all of the Fe adsorbed on the column while in its presence, a peak appeared representing ∼27% of the absorption area of an Fe-citrate standard containing the same concentration of Fe. These results suggested that citrate at physiological concentration might coordinate as much as 27% the iron that would otherwise form the Fe^III^ aggregate. However, sodium citrate by itself chelated some adsorbed iron from the column, so 27% is an upper limit. In the same experiment, sodium citrate was passed through the column before (Citrate 1) and after (Citrate 2) the other samples. The higher intensity after the other samples were run reflected the adsorption of iron from those other samples and the ability of citrate to remove some of the adsorbed iron. Thus, some of the 27% Fe^III^ citrate peak intensity was due to citrate’s ability to remove iron from the column.

Higher concentrations of citrate were able to prevent Fe^III^ aggregation to a greater degree. When an extremely high concentration citrate (2500 μM, ca. 25 × physiological) was included in pseudo plasma, followed by 250 μM ^57^Fe^III^, Mössbauer spectra showed that the added iron did not aggregate (Fig. S6). When 500 μM citrate (5 × physiological) was used, some ^57^Fe^III^ aggregated, and some formed an uncharacterized magnetic species (perhaps an intermediate). We are uncertain whether the products formed in these experiments were kinetically or thermodynamically controlled, but they indicate the variety of outcomes depending on the concentration of citrate present in pseudo (and real) plasma. Under physiological conditions (ca. 100 μM citrate), the Fe^III^ aggregate will dominate or exclusively constitute the non-TFN non-FTN (nonproteinaceous) fraction of plasma iron.

**Figure 9:**
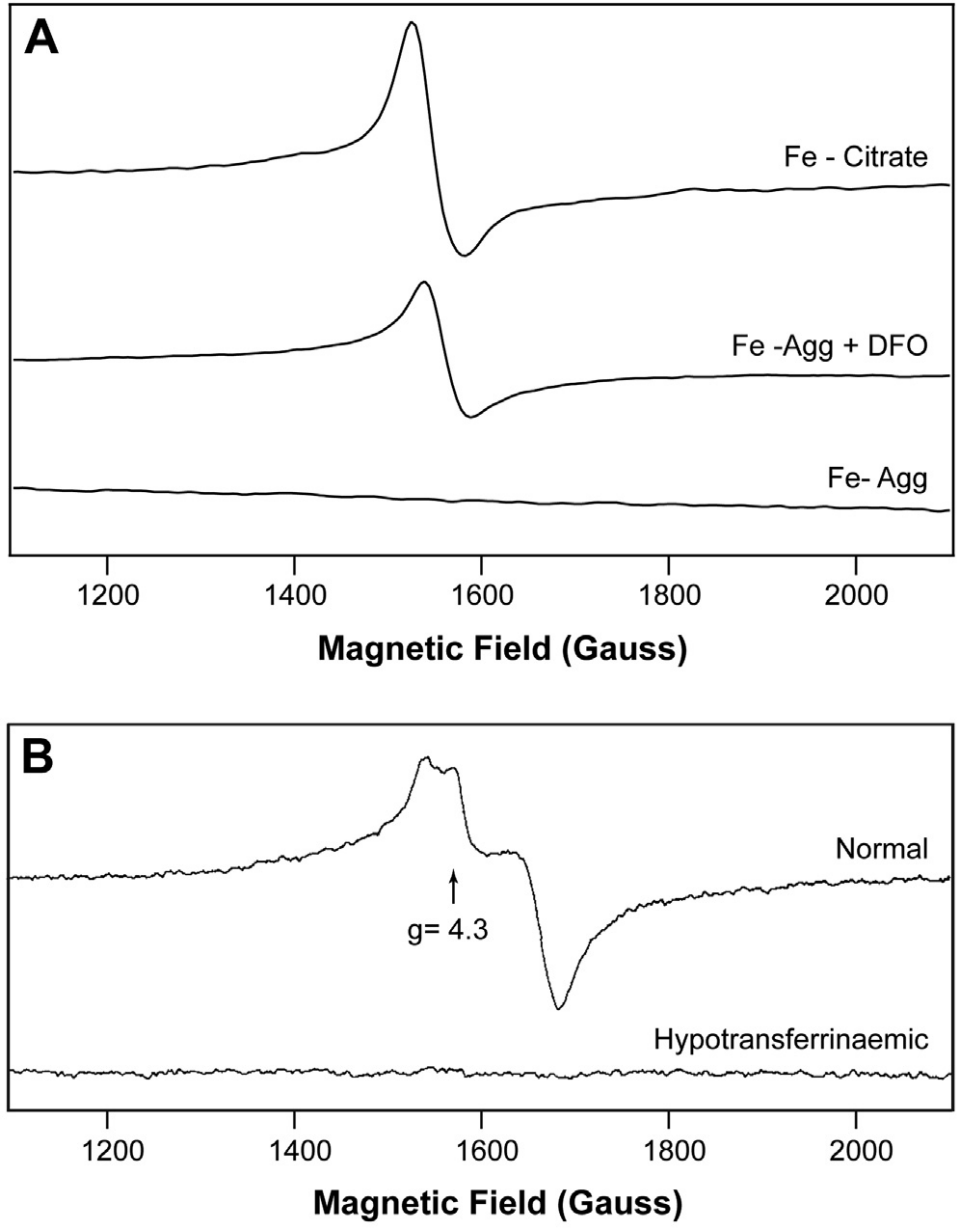
**X-band EPR spectra of plasma-related iron species.***Panel A*, *top*, Fe^III^ citrate standard from Figure 7*B*; *middle*, Fe^III^ in pseudo-plasma treated with DFO, from Figure 7*G*; bottom, Fe^III^ in pseudo-plasma, from Figure 7*E*. Parameters: microwave frequency, 9.354 GHz; temperature, 11K; microwave power, 0.02 mW, modulation amplitude, 10 G; modulation frequency, 100 kHz; sweep time, 120 s; 4 scans averaged. *Panel B*, EPR spectra of sera isolated from normal and hypotransferrinemic mice. Taken from Simpson *et al.* (27) with permission from the publisher. EPR, electron paramagnetic resonance; DFO, deferoxamine.

Finally, we thawed the MB samples used to generate Figure 7, *B*, *E* and *G*, and transferred them to electron paramagnetic resonance (EPR) tubes. The Fe^III^ citrate sample exhibited a g = 4.3 EPR signal (Fig. 9, Panel A, top spectrum), similar to those previously reported (11, 27). Such signals arise from S = 5/2 states with rhombic symmetry. In contrast, the Fe^III^ aggregate was EPR-silent (Fig. 9*A*, bottom spectrum), as expected for magnetically interacting Fe^III^ nanoparticles. The spectrum of the DFO-treated sample (Fig. 9*A*, middle spectrum) exhibited a g = 4.3 signal that was about half the intensity of the Fe^III^ citrate signal, consistent with the corresponding MB spectrum (Fig. 7*G*). All three samples contained 250 μM iron.

## Discussion

In the 1960s, all nonheme iron in serum was generally thought to be bound to TFN, though some experiments were suggesting otherwise (6). In 1970, Sarkar (7) titrated sera with ^59^FeCl_3_ and removed the TFN-bound fraction by ultracentrifugation. Early in the titration, essentially all added ^59^Fe bound to apo-TFN, but once this protein became saturated, a significant portion of ^59^Fe remained in the protein-free supernatant. This suggested that sera contained a LMM ligand (:L_NTBI_) capable of binding the excess added ^59^Fe. After attempting to remove this ligand by dialysis, the titration behavior could only be replicated by adding citrate to dialyzed sera, highlighting the possibility that citrate was the sought-after :L_NTBI_ ligand. Viewed from our current perspective, the results of Sarkar imply that endogenous NTBI is a high-mass species that can be mobilized by coordination with citrate to form a low-mass complex. Sarkar suggested that the high-mass species was/were iron-binding proteins other than TFN. Although not considered at the time, an Fe^III^ aggregate would also be consistent with his results.

In 1978, Hershko *et al.* (8) connected NTBI to iron-overload diseases. They detected a chelatable form of iron (2–7 μM), not bound to TFN, in sera of patients with β-thalassemia that was absent in controls. They found that the NTBI concentration was related to the degree of TFN saturation past a threshold and that NTBI could be pried from its ligand with strong chelators or apo-TFN. A year later, Graham *et al.* (9) quantified NTBI by adding EDTA to sera from thalassemia patients and passing the solution through a 25 kDa cutoff membrane. In the absence of EDTA, the FTS lacked NTBI, again suggesting that NTBI was a high-mass iron species that could not pass through the membrane unless mobilized by a chelator. Based on these considerations, Graham *et al.* concluded like Sarkar, that NTBI was “bound to serum proteins”. However, their results are also consistent with NTBI being a high-molecular-mass nonproteinaceous Fe^III^ aggregate that could be mobilized by chelators like EDTA, citrate, and DFO, etc.

In 1980, Batey *et al.* (10) reported that NTBI adheres strongly to DEAE-Sephadex chromatography columns and used this “stickiness” to determine the percentage of serum iron that was NTBI. They added ^59^Fe^III^ citrate which preferentially bound apo-TFN, but once TFN was saturated, the added ^59^Fe^III^ citrate adhered to the column (perhaps some of the added complex converted to ^59^Fe^III^ aggregates).

Many subsequent NTBI studies focused on standardizing an NTBI assay and quantifying the concentration of the NTBI pool, as obtained by incubating sera or plasma with various chelators under different reaction conditions (28). Other studies investigated the NTBI = Fe^III^ citrate hypothesis. In 1987, Craven *et al.* (29) injected ^59^Fe citrate into genetically modified hypotransferrinemic mice (which have low TFN levels) and controls. They found that in these mutant mice, nearly all of the injected ^59^Fe that was absorbed by the body localized in the liver and pancreas. This reinforced the idea that NTBI was Fe^III^ citrate. Perhaps some of the added Fe^III^ citrate may have been converted to an Fe^III^ aggregate once in the blood.

Our current results reinforce our earlier conclusion that the FTS of blood from both healthy and hemochromatosis individuals contain minor amounts of iron that could be NTBI (20, 21). Although the source of iron giving rise to these minor species remains unknown, our current results indicate that the concentrations of these species are independent of the concentration of iron in the diet. The idea that NTBI might not be a LMM species was prompted by our realization that only the column used to run retentate fractions was becoming fouled with excess iron. Unfortunately, this realization occurred toward the end of the study, limiting the follow-up experiments that could be performed.

Probing Fe aggregation in pseudo plasma using LC-ICP-MS showed that citrate at high concentrations can inhibit formation of the Fe^III^ aggregate. However, the required citrate concentration is higher than the typical range of citrate concentrations in blood plasma. This suggests an equilibrium {Fe^III^ aggregate + citrate ⇄ Fe^III^citrate + nH_2_O + other nonproteinaceous ligands}, with the Fe^III^ aggregate dominating under physiological conditions. Whether the distribution (Fe^III^ aggregate *versus* Fe^III^ citrate) is kinetically or thermodynamically controlled in blood plasma remains uncertain. A similar equilibrium involving TFN {Fe^III^ aggregate + apo-TFN → TFN + nH_2_O etc.} is probably also likely but with rapid kinetics and thermodynamics favoring products.

**Figure 10:**
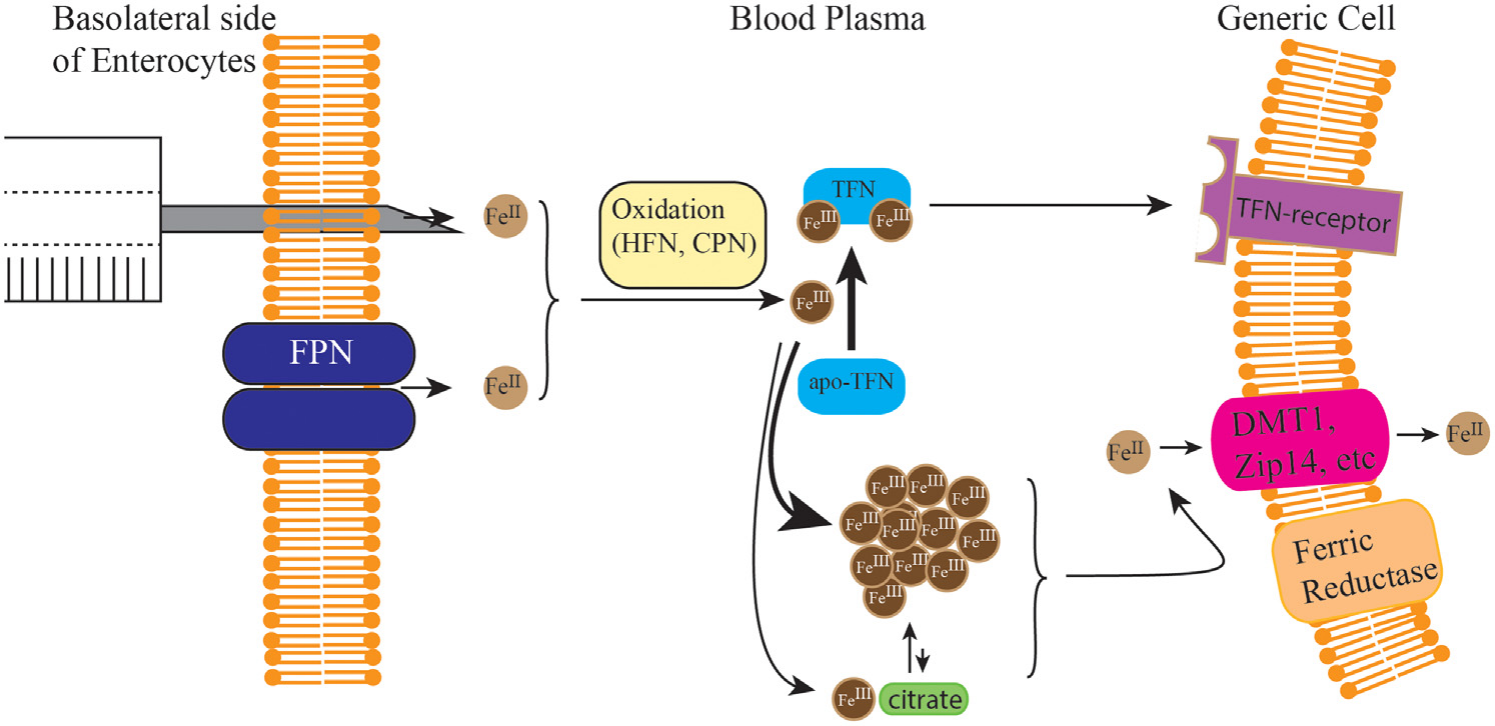
**Model for the formation and metabolism of NTBI.** See text for details. CPN, ceruloplasmin; FPN, ferroportin; HFN, hephaestin; NTBI, nontransferrin-bound iron; TFN, transferrin.

According to our model (Fig. 10), bare Fe^II^ ions enter the blood through FPN and are quickly hydrated and oxidized by hephaestin or ceruloplasmin. A similar result would be obtained if exogenous Fe^II^ or Fe^III^ salts were injected into the blood *via* syringe. If apo-TFN was available, the newly formed Fe^III^ ions preferentially coordinate to this protein, but when none is available (saturated or nearly so), most will aggregate to form a high-molecular-mass species that is retained by 10 kDa cutoff membranes and adheres readily to chromatography columns. Entry of NTBI into bodily cells requires prereduction to the Fe^II^ state followed by entry *via* Zip14, DMT1, or other Fe^II^ importers (3).

Grootveld *et al.* (30) is typically cited as strong evidence that NTBI = Fe^III^ citrate. However, we find this conclusion only partially supported by their data. These authors examined plasma and plasma ultrafiltrates (equivalent to our FTSs) from hemochromatosis patients and healthy controls, using NMR spectroscopy and reverse-phase HPLC. Hemochromatosis plasma contained an average of 370 ± 60 μM citrate, ∼3 × higher than in healthy controls. They detected corresponding NMR spectra of hemochromatosis plasma with signals due to unbound citrate (high-spin Fe^III^citrate is undetectable due to paramagnetic line broadening). They reported that the NTBI concentration in such plasma was ∼5 μM Fe and that adding DES resulted in “small increases” in citrate NMR intensities. They assumed that this increase arose from the chelation reaction {Fe^III^citrate + DES → Fe^III^DES + citrate}. However, according to concentrations that they reported, complete coordination of citrate in hemochromatosis plasma would have only increased the NMR citrate peak intensities by < 2%, well within reported uncertainties. Also, DES treatment affected the intensity of other NMR signals (*e.g.*, from acetate) to an even greater extent. This implies that the observed increase in NMR intensity was due to causes other than the suggested reaction, and they do not lend support to the NTBI = Fe^III^citrate hypothesis. The NMR spectra actually imply that most citrate in plasma is not coordinated by Fe^III^.

In another experiment, Grootveld *et al.* added 100 to 500 μM FeCl_3_ to plasma in a titration monitoring the free citrate NMR peaks and observed a gradual broadening of these peaks. They reasonably concluded that broadening arose from the binding of the added Fe^III^ to free citrate, but the concentration of Fe used was 20 to 100 × higher than physiological and so does not seem relevant to actual conditions in blood.

They also performed LC studies of plasma and ultrafiltrates (equivalent to our FTS). In one LC experiment (Fig. 8; (30)), a peak due to ferrioxamine (Fe^III^-DES) was obtained when hemochromatosis plasma ultrafiltrate was treated with DES, from which they reasonably concluded that DES mobilizes (and coordinates) NTBI. However, those results do not impact whether NTBI = Fe^III^ citrate.

In other LC experiments, plasma FTS from healthy controls exhibited a minor shoulder (at 8.25 min; Fig. 5*A*; (30)) that comigrated with Fe^III^ citrate, and its intensity increased slightly in traces of hemochromatosis FTS or in control FTS when FeCl_3_ was added (Fig. 5*C*; (30)). These results support the NTBI = Fe^III^ citrate hypothesis, but concerns remain. They used a column in which polar hydrophilic species like Fe^III^ citrate are eluted unresolved at the void volume possibly along with other 340 nm-absorbing species. Thus, the intensity of the peak at 8.25 min may not be solely due to Fe^III^ citrate. Second, their elution profiles of plasma FTS exhibited numerous features with intense “negative absorption” which is physically impossible; the problem was not mentioned.

May *et al.* (18) is another often cited study supporting the NTBI = Fe^III^ citrate hypothesis. In their recent review, Silva and Rangel (14) correctly reported that May *et al.* predicted that 99% of iron in plasma was associated with citrate (we assume they meant 99% of non-TFN–bound plasma iron). However, left unmentioned was that May *et al.* also predicted that the total low-molecular-weight *iron concentration* in plasma should be 6.68 × 10^−13^ M (see Table 5 of May and Linder (18)), ca. 7 orders of magnitude lower than the estimated size of the NTBI pool.

The tendency of ferric ions to form high mass aggregates in aqueous solutions at neutral pH is well known. Such nanoparticles can have masses of ∼ 150 kDa (Ref 11 and references therein). Using the *K*_*sp*_ of ferric hydroxide and *K*_*eq*_’s for competing iron binding reactions, Evans *et al.* found that Fe^III^ hydroxide dominates under equilibrium conditions. However, they found that the precipitation reaction was slow and concluded that the aggregate would not dominate in plasma where product distributions would be kinetically controlled. They considered that the high mass NTBI species might be Fe^III^ bound to serum albumin but ultimately concluded that binding was weak and easily disrupted. A later study by Silva and Hider (31) suggested the NTBI might be a heterogenous mixture of Fe^III^:albumin and Fe^III^ citrate. Silva and Rangel (14) suggested that Fe^III^ in plasma might bind nonspecifically to the 98 carboxylate groups on the surface of albumin. Their model of NTBI assumes binding to citrate and serum albumin in a fine-tuned equilibrium depending on Fe^III^ concentration, the iron:citrate molar ratio, and albumin posttranslational modifications.

### The NTBI = Fe^III^ aggregate hypothesis

Our results and evaluation of the NTBI literature support the idea that NTBI is an Fe^III^ aggregate composed of magnetically interacting Fe^III^ ions. Further studies are required to determine the exact chemical composition of this material. Although the size of the aggregated particles is also uncertain, they appear large enough to be retained by a 10 kDa cutoff membrane filter. These aggregates also adhere to our LC column and can be mobilized by Fe^III^ chelators like DFO or citrate (but only at high concentrations). We suspect that the nanoparticles dissolve upon reduction, allowing Fe^II^ chelators to become effective.

Our last line of evidence for this hypothesis comes from EPR spectra of sera from WT and hypotransferrinemic mice (27) in conjunction with spectra of Fe^III^ citrate and other standards (11). Spectra of sera from WT mice are dominated by a g = 4.3 signal which arises from high-spin Fe^III^ ions bound to TFN that are not magnetically interacting. In Figure 9*B*, top spectrum, we have reproduced the published spectrum of Simpson *et al.* (27). Similar g = 4.3 signals are observed for Fe^III^ citrate, Fe^III^ albumin, and Fe^III^ DFO. In contrast, EPR spectra of sera from hypotransferrinaemic mice, which lack TFN, are devoid of a g = 4.3 signal (27), identical to what we obtained using an Fe^III^ aggregate sample (Fig. 9). Their spectrum is reproduced in Figure 9*B*, bottom. Simpson *et al.* (27) suggested “that the majority of the iron (in sera of these TFN-deficient mice) is polynuclear” as would be the case for our NTBI = Fe^III^ aggregate hypothesis. The lack of g = 4.3 signal also excludes the presence of (high concentrations of) Fe^III^ citrate or Fe^III^ albumin in sera; such species would also exhibit g = 4.3 signals. Evans *et al.* (11) found that Fe^III^ citrate solutions were devoid of g = 4.3 signals when the Fe:citrate ratio was 1:10 and 1:100 but that the signal developed when ratios were increased to 1:1000 and then 1:10,000. They concluded that “oligomeric and polymeric species containing oxygen-bridged iron(III) ions” were present at low iron:citrate ratios and that this was converted to magnetically isolated complexes at higher ratios. This suggests that Fe^III^ aggregates shift to mononuclear Fe^III^ citrate complexes as the concentration of citrate increases. All of these past results support and confirm the results presented here as well as our hypothesis regarding the nature of NTBI. Additional studies are being planned to test our hypothesis further. We hope that these insights and the eventual chemical identification of NTBI will help in developing new treatments for iron-overload diseases.

## Experimental procedures

All procedures involving mice were approved by the Animal Use Committee at Texas A&M University (AUP 2018-0204). *HFE* mice (stock number 017784, B6.129S6-Hfe<tm2Nca>/J) and control mice (C57BL/6J) were purchased from The Jackson Laboratory (www.jax.org). Animals were housed in the LAAR facility in the School of Veterinary Medicine at Texas A&M University. Mice were raised in disposable all-plastic cages (Innovive model MVX1) containing synthetic bedding (Alpha-Dri Irradiated; Lab Supply, Houston) and all-plastic water bottles. Room temperature was 74 ± 2 °F. Lighting was on a 12/12 h cycle. Mice were initially bred on an iron-deficient mouse diet (TD.80396.PWD; www.envigo.com) spiked with 0, 50, 500, and 5000 mg of Fe^III^(citrate) per kg chow. Each diet was prepared by weighing out the appropriate mass of FeSO_4_ dissolved in solutions of 10 × excess sodium ascorbate and mixed with the Fe-deficient chow powder. The resulting moistened material was pelleted manually by pressing into a plastic pipe with a tight-fitting glass rod. Pellets were baked in a glass pan at 75 °C for 4 to 7 h and then refrigerated until used. Mice were offered food and distilled water *ad libitum*. Only breeding pairs given the 50 mg/kg diet bred successfully; so upon weaning, mice from that food group were switched to a designated diet to balance the study.

Animals ranging from 3 to 52 weeks were transported to the Chemistry department at TAMU where they were sacrificed. Mice were anesthetized by injecting ketamine (5 mg/20 gm mouse) and xylazine (1 mg/20 gm mouse) subcutaneously. Exsanguination was by cardiac puncture once tests for pain (foot-pad squeeze) showed no response. Between 0.5 and 1.2 ml of blood was removed from each animal. Blood samples from two animals grown on the same diets were routinely combined. Samples were spun by centrifugation (2500×*g* for 15 min using a Sorvall RC centrifuge) and imported into a refrigerated N_2_-atmosphere glove box (Mbraun Labmaster 130) containing 1 to 20 ppm O_2_. Plasma was collected by syringe, transferred to epi-tubs, removed from the box, and stored at −80 °C.

### Liquid chromatography-inductively coupled plasma mass spectrometry

FTSs and retentate fractions were obtained from plasma using Amicon Ultra (2 ml) 10 kDa filters (Millipore). Five hundred microliters of plasma sample was loaded onto the activated filter and centrifuged at 2500×*g* until ∼ 400 μl solution was collected as the FTS (25–60 min). The retained fraction (retentate) was diluted using 20 mM ammonium acetate buffer to the original volume of the plasma. FTSs and retentates were passed through a 0.2 μm filter (to remove red blood cells and any debris) and then injected on the LC-ICP-MS system which consisted of an Agilent Bioinert LC housed in an anaerobic glovebox at 5 to 10 °C interfaced with an online ICP-MS (Agilent 7700×). Two size-exclusion chromatography columns were used, including Superdex 200 C and Superdex Peptide 10/300 Gl (Cytiva). These are referred to as the “high-mass” and “low-mass” columns, respectively. Columns were equilibrated with filtered and degassed 20 mM ammonium acetate, pH 6.5, at a flow rate of 0.6 ml/min. In later experiments, retentate fractions were diluted 80× using 20 mM ammonium acetate and then analyzed by LC-ICP-MS. ^56^Fe and/or ^57^Fe were detected in He collision mode with a dwell time of 0.1 s. A ‘ghost column’ composed of PEEK tubing replaced actual columns to assess the total iron present in the samples and indirectly the portion adsorbed on actual columns.

### Mössbauer spectroscopy

Retentate fractions from *HFE* mice raised on diets containing 50 mg of ^57^Fe-enriched per kg chow were combined based on age. The “young” sample combined retentates from 4-, 6-, 10-, and 14-weeks-old mice; the “old” sample combined retentates from 18, 24, 32, and 48 weeks. Each sample (∼2 ml) was concentrated using 10 kDa cutoff Amicon Ultra filters to a final volume of 400 to 500 μl. These were transferred to MB cups and frozen in LN_2_ until analyzed. Unlike the LC-ICP-MS experiments, MB samples were not passed through a 0.2 μm filter and so they had minor red-blood cell contamination.

Pseudo plasma was prepared as a 2 × stock of plasma salts, including 5 mM potassium chloride (all final 1 × concentrations), 28 mM sodium bicarbonate, 1.45 mM potassium phosphate, 0.1 g/L calcium chloride, 1 mM magnesium chloride, and 112 mM sodium chloride, adjusted to pH 7.4 with HCl. For some preparations, bovine serum albumin (Pierce) and sodium citrate were added at final concentrations of 0.2 g/L and 150 μM, respectively. Acidic ^57^Fe^III^ was prepared by dissolving 1.00 g of ^57^Fe_2_O_3_ (Cambridge Isotope Laboratories; 95.5% enrichment) in 3 to 5 ml of concentrate HCl followed by heating and then dilution to 40 mM final Fe concentration. Solutions were stored at 4 °C. The ^57^Fe stock was mixed with pseudo-plasma salts, yielding 1 × plasma salts and 250 μM ^57^Fe final concentrations. Samples were frozen in MB cups after a 1 h incubation on the bench top at room temperature. Mӧssbauer spectra were collected on an MS4 WRC spectrometer (SEE Co) at 5 to 6 K with a field of 0.05 T applied parallel to the gamma radiation. Instruments were calibrated at room temperature with α-Fe foil. Spectra were simulated using WMOSS (http://www.wmoss.org/) software.

### Metal analysis using ICP-MS

FTS from six mice were used to prepare three replicates of samples by combining two FTS at a time. The samples were digested with 2 × volume of trace-metal-grade HNO_3_ (Fisher) at 70 °C for 16 h in sealed plastic falcon tubes. Following digestion, samples were cooled to room temperature and diluted to obtain a 5% final HNO_3_ concentration. Metal content was analyzed with ICP-MS. A series of five ICP-MS iron calibration standards were prepared with a custom-made TEXASAM-15REV3 stock (Inorganic ventures). The concentrated stock contained 1 mg/L of natural abundance Fe. The remaining standards were obtained by diluting the previous standard 10×.

### Figure 5 experiment

Plasma from 36-weeks-old regular *HFE* mice (∼500 μl) was concentrated using a 10 kDa cutoff filter, and FTS was collected. To wash the sample, 450 μl of water was added to the ∼ 50 μl of the retentate. The sample was again concentrated, and FTS collected. The washed retentate was diluted 80× with 20 mM ammonium acetate pH 6.5 and divided in half. Half was treated with 50 μM sodium citrate, and half was untreated. A similar experiment was performed using plasma from 24-weeks-old regular *HFE* mice. In another experiment, FTSs from six 24 to 36 weeks *HFE* mice (0, 50, 500, and 5000 mg) were combined and then divided in half. Half was treated with 50 μM Na citrate, and half was untreated.

## Data availability

All data are contained within the manuscript and SI.

## Supporting information

This article contains supporting information.

## Conflict of interest

The authors declare that they have no conflicts of interest with the contents of this article.
