# Supplementary material for: Might nontransferrin-bound iron in blood plasma and sera be a nonproteinaceous high-molecular-mass FeIII aggregate?
Source: J Biol Chem. 2022 Nov 9;298(12):102667. doi: 10.1016/j.jbc.2022.102667 (PMC9768373; doi:10.1016/j.jbc.2022.102667)

Supplemental Information for:

Might non-transferrin-bound iron in blood plasma and sera be a non-proteinaceous high-molecular-mass Fe^III^ aggregate?

Shaik Waseem Vali^1^ and Paul A. Lindahl^1,2^*

Figure S1 Flowchart of mice utilization in the study

Figure S2 LC-ICP-MS chromatograms of FTS from different batches run on the low-mass column.

Figure S3 LC-ICP-MS chromatograms of retentates from HFE and control mice.

Figure S4 LC-ICP-MS of control and HFE retentates run on the low-mass column.

Figure S5 Mössbauer spectra of pseudo-plasma salts with added Fe-citrate.

Figure S6 Raw Mössbauer spectra of HFE retentate plasma before subtracting oxy- and deoxyhemoglobin contributions.

Figure S1: Flowchart of experiments


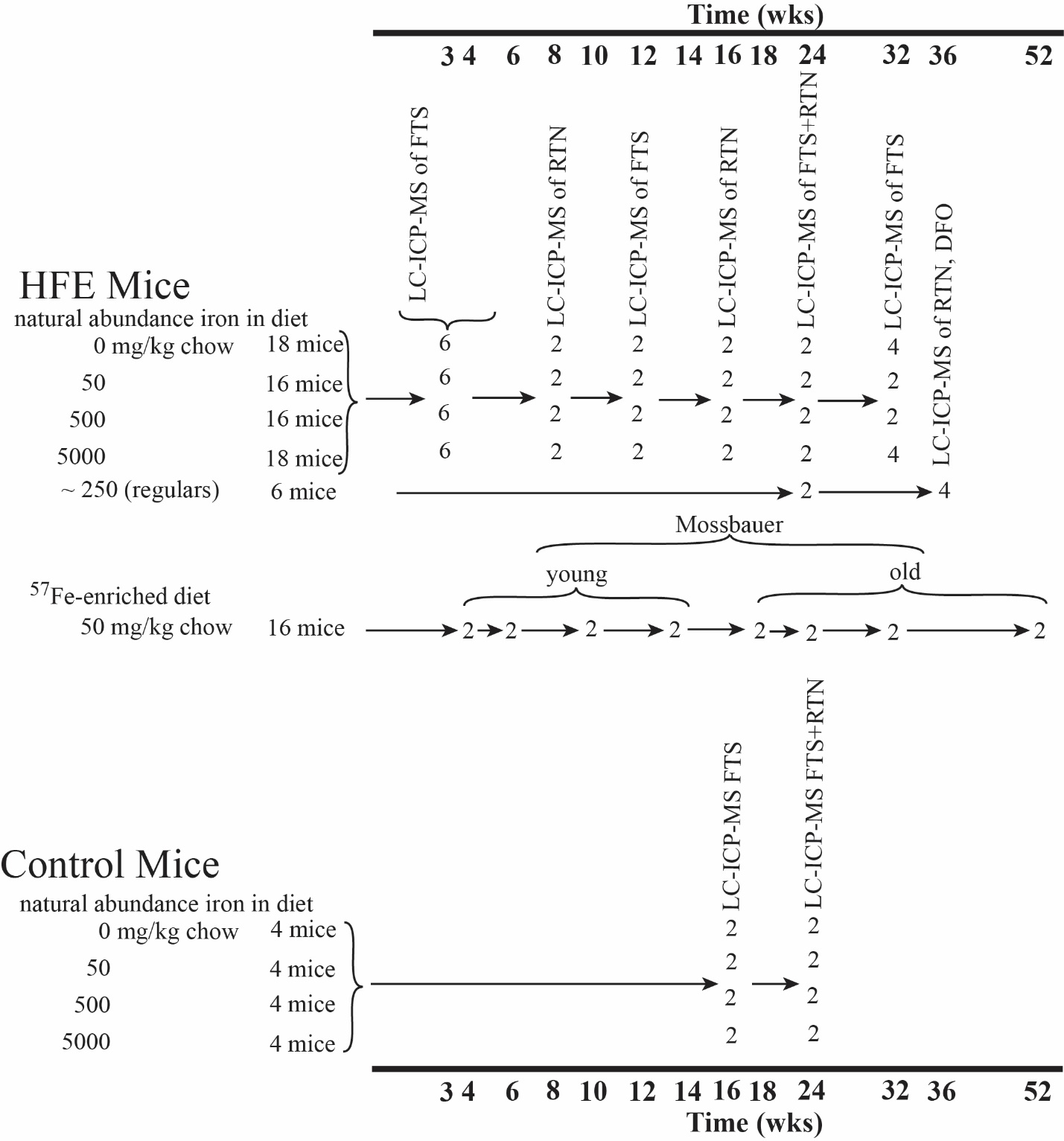


**Figure S2: LC-ICP-MS chromatograms of FTS from different batches run on the low-mass column.** A (32 week), B (3 week), C (24 week) are FTS from HFE mice. Red line in C shows an Fe citrate standard (2 µM Fe). D, FTS from 24-week-old control mice and blue traces indicate sample run through a tubing (ghost column) to determine the actual iron peak in the sample as opposed to contamination from the column. No significant difference between 0 mg and 5000 mg samples was observed within each group.


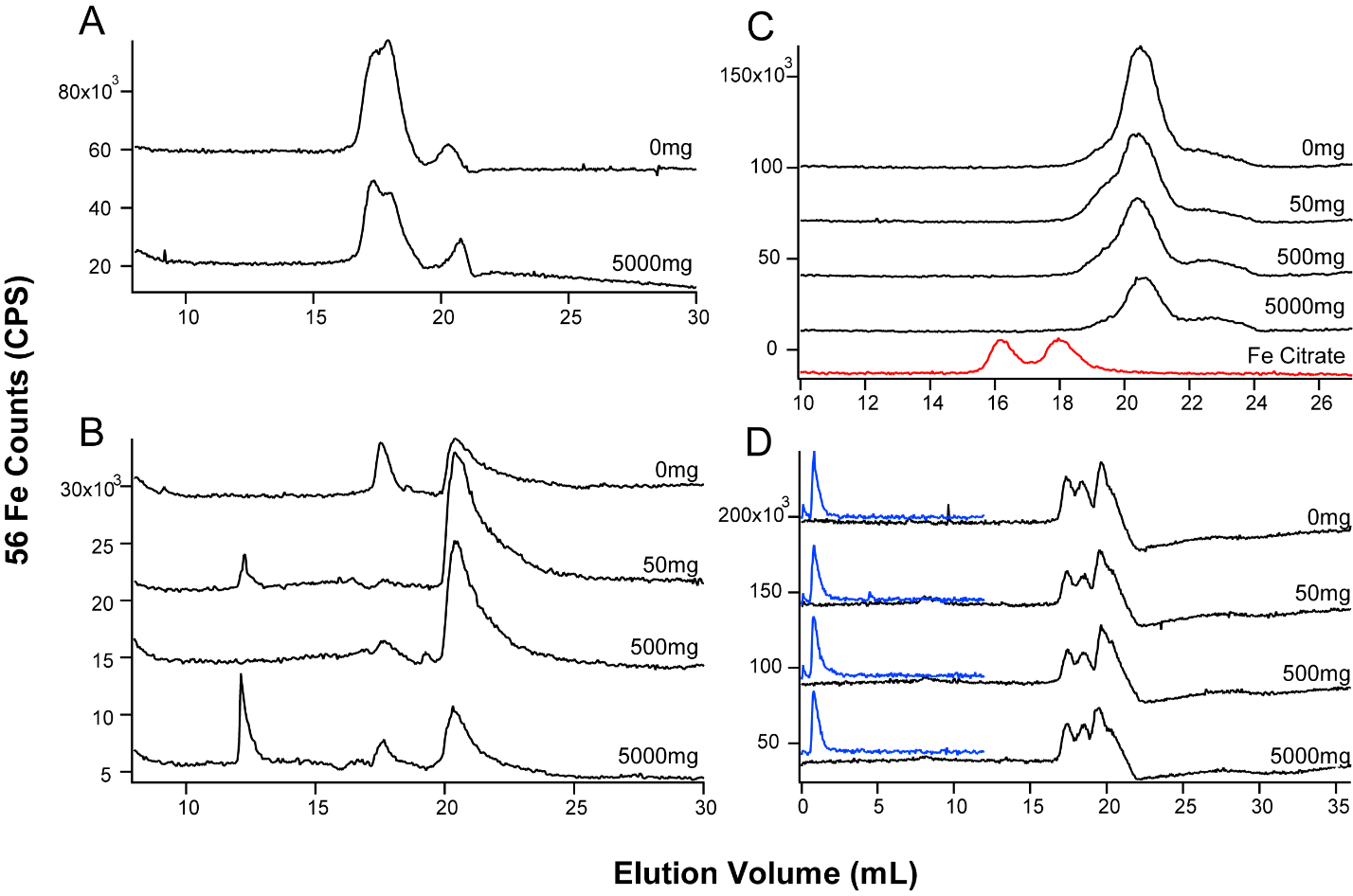


**Figure S3: LC-ICP-MS traces for retentates from HFE and control mice.** A, 8-week-old HFE mice retentate; B, 16-week-old HFE mice retentate; C, 24-week-old HFE mice retentate and D, 15-week-old control mice retentate. The traces show two major peaks assigned to serum ferritin (FTN) and transferrin (TFN). Samples were analyzed using the high-mass column, with 20 mM ammonium acetate pH 6.5 mobile phase at 0.5 ml/min flow rate.


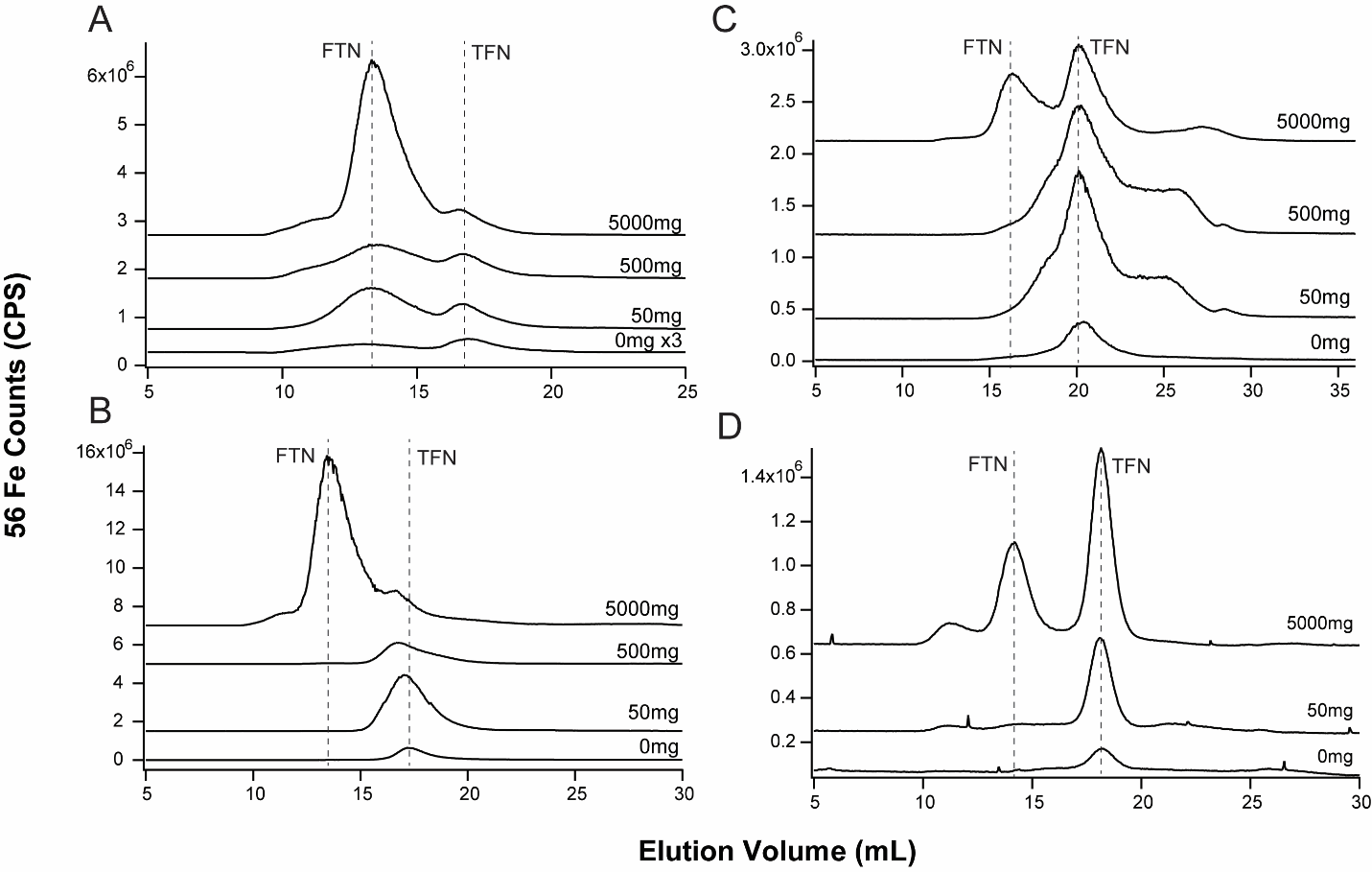


**Figure S4: LC-ICP-MS of control and HFE retentate run on the low-mass column.** The lack of peaks in the resolvable region indicates that all iron species either eluted at the void volume as high-molecular mass species (including TFN and FTN) or adhered to the column.


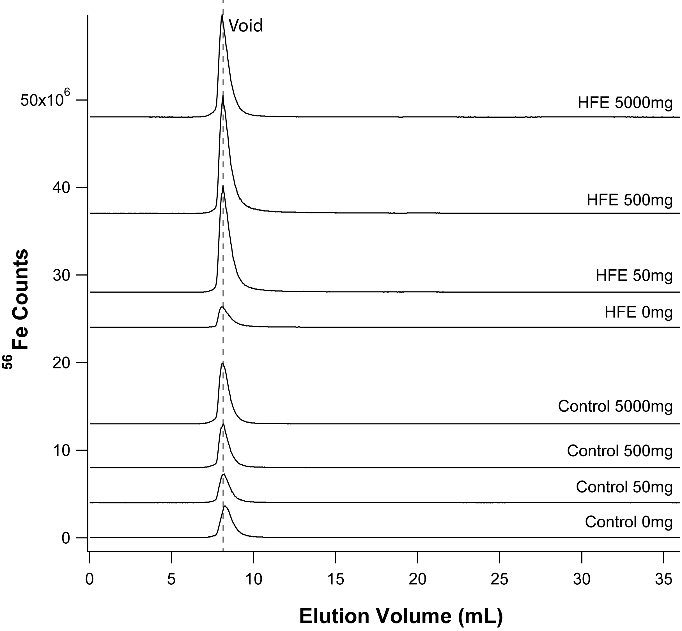


**Figure S5: Raw 5K Mössbauer spectra of retentate plasma before subtracting signal from oxy- and deoxyhemoglobin.** A, 4, 6, 10, and 14 weeks old retentates combined, concentrated using a 10 kDa cutoff membrane, loaded into a MB cup and frozen in LN2. B, 18, 24, 32, and 48 weeks old retentates combined and treated similarly. The parameters for deoxy (1) and oxy (2) hemoglobin doublets (red brackets) were δ_1_ = 0.93 ± 0.02 mm/s; ΔE_Q1_ = 2.35 ± 0.03 mm/s and δ_2_ = 0.29 ± 0.02 mm/s; ΔE_Q2_ = 2.30 ± 0.03 mm/s. Linewidths were 0.35 mm/s.

**
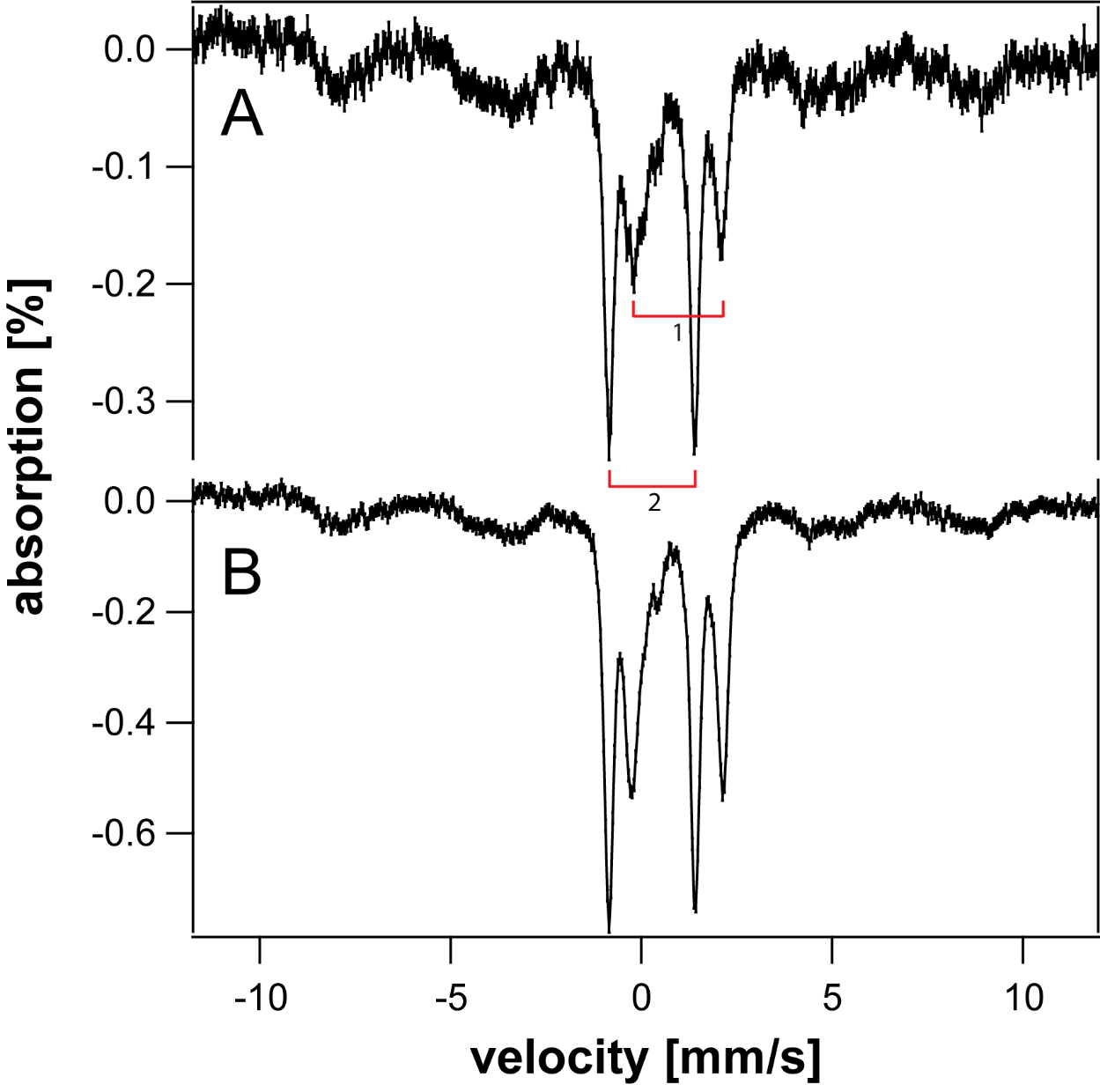
**

**Figure S6: Mössbauer spectra (5 K, 0.05 T, parallel) of pseudo-plasma salts with added Fe-citrate.** A, Fe citrate (250 μM Fe, 2.5 mM citrate) added to plasma salts followed by incubation at RT on bench top for 1 h prior to freezing in liquid N_2_; B, Fe citrate (250 µM Fe, 500 mM citrate) added to plasma salts followed by incubation at RT on bench top for 1 h prior to freezing.


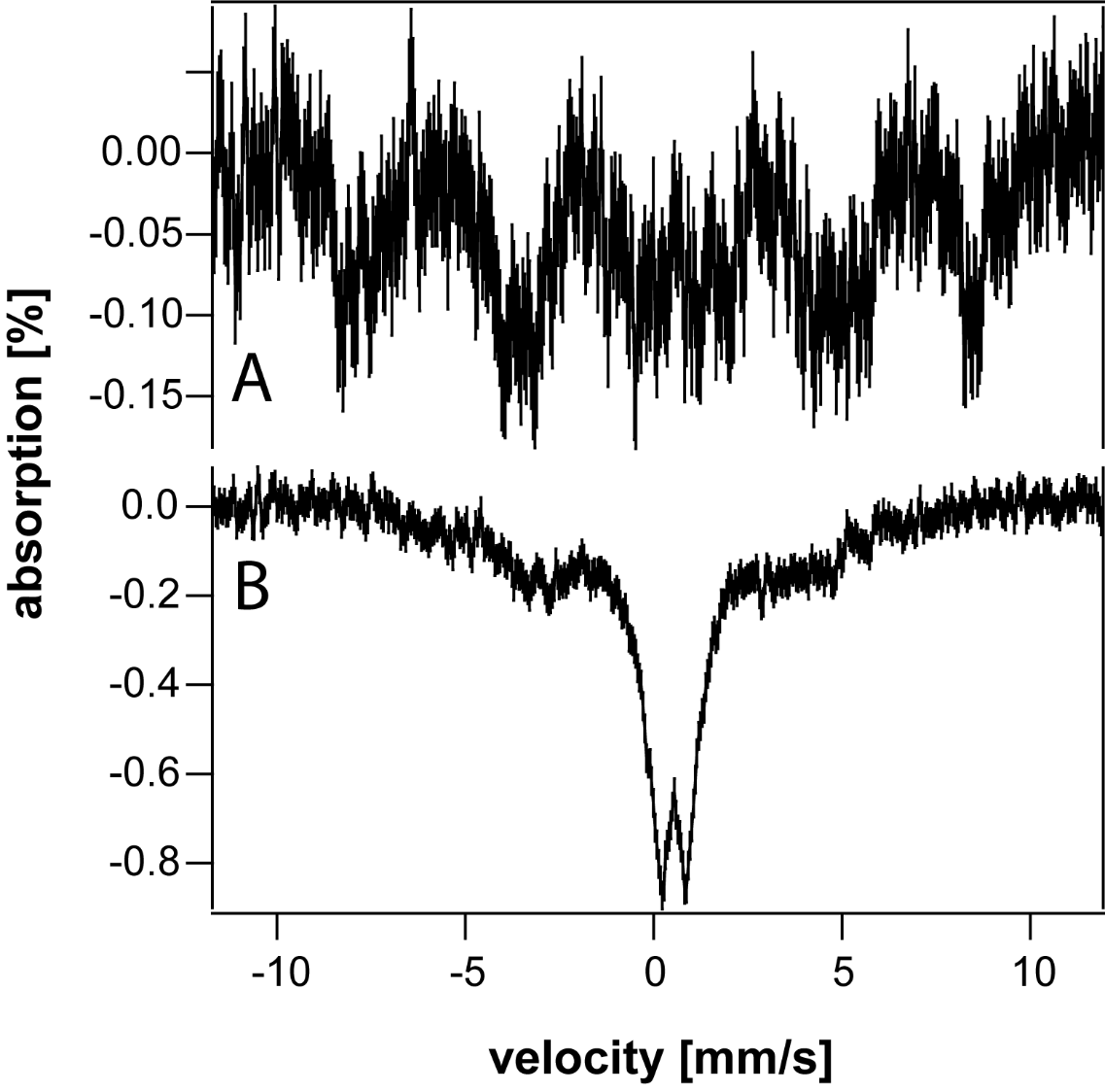

Supplement: Supplemental Figure [file mmc1.docx]
